# Assessing and measuring community health system resilience – an updated scoping review of approaches

**DOI:** 10.1186/s12913-025-13802-6

**Published:** 2025-12-04

**Authors:** Johannes Espmark, Dell D. Saulnier, Robert Šakić Trogrlić, Sharif A. Ismail

**Affiliations:** 1 Department of Global Public Health, Karolinska Institutet, Stockholm, Sweden; 2 International Institute for Applied Systems Analysis, Laxenburg, Austria; 3 Department of Global Health and Development, London School of Hygiene and Tropical Medicine, London, UK

**Keywords:** Resilience, Community, Health system, Scoping review

## Abstract

**Background:**

A previous 2020 review on resilience in community health systems identified a small body of conceptual and empirical evidence but pointed to important limitations concerning the operationalisation of resilience assessment and measurement for these systems, and lack of consideration to measures of equity. In light of substantial research interest in health system resilience over recent years, the objective of this study was to revisit the literature for new insights concerning community health system resilience assessment and measurement in general, and in relation to climate-related hazards in particular.

**Methods:**

Scoping review of published, peer-reviewed literature drawing on studies identified via keyword-structured searches of PubMed and Google Scholar covering the period 2019–2024. Following screening in duplicate, we included studies from all income settings that provided definition(s) of community health system resilience. Data were extracted in duplicate and narratively synthesised drawing on a conceptual framework from Disaster Risk Management to identify putative approaches to resilience assessment and/or measurement – including candidate metrics.

**Results:**

12 studies were included, of which 5 explicitly focused on natural and/or climatic hazards. Definitions of community resilience were diverse, spanning process and attribute-based conceptualisations among others. Included studies yielded a total of 73 resilience indicators in addition to those 20 identified through the original scoping review. A large majority of indicators (*n* = 51) spanning both reviews covered background factors relating to community resilience such as markers of community social capital and local health system capacity. Other indicators considered, in order of frequency, community preparedness, event response and post-event recovery – although this last category included by far the fewest indicators. Equity considerations were often implicit rather than explicit within these – commonly focusing on equity in health service access at community level, and degree of participation in governance processes.

**Conclusions:**

There is a continuing need to develop – and particularly to empirically test – indicators of community health system resilience to better understand utility for policy and practice. While some promising areas are identifiable for development of indicators relating to equity, these remain early stage and there is a need to better conceptualise links between these and established health system outcome measures.

**Supplementary Information:**

The online version contains supplementary material available at 10.1186/s12913-025-13802-6.

## Introduction

Climate-driven hazards continue to cause significant harm to health and health systems. Health systems must continually adapt to the impacts, such as short- and long-term variations in health service demands, to the physical and economic damage to the health system and to local communities from disasters and extreme weather events, and to changing population and community risks [1]. Building health system resilience, defined here as the capability to absorb, adapt, and/or transform so that essential and routine health functions are maintained during a shock or during periods of chronic stress [2, 3], is one approach to managing the impacts of climate change [4].

Local communities at risk play a central role in building resilience and are typically the first actors to respond to a climatic event [5]. Despite broad acknowledgement of both these facts and of the inequitable distribution of impacts from climatic hazards and other shocks or stressors on at-risk population groups [6–10], links between community-level activities and broader system resilience are under-theorised [11]. A health system’s capacity for resilience is partly determined by interactions among health system actors and with other systems, such as communities, and the context where they exist [12, 13]. Linked community and health system factors such as trustworthiness, legitimacy, and power structures can all influence a system’s ability to manage a shock or stress [14–16]. A community’s own capacity for resilience may also influence how health systems prepare for or respond to shocks and stresses, or the absorptive, adaptive, and transformative changes it undergoes. For example, community health workers and volunteers were a main pillar to Covid containment efforts globally [17], and community dynamics appeared to underlie transformative change in Mauritania during Covid [18].

Importantly, resilience is a dynamic concept. Existing literature captures the dynamic nature of resilience in different ways, but often with a focus on resilience strategies including absorption, adaptation and transformation [19]. Within the broader field of disaster risk management, dynamism is often captured through cyclical representations encompassing a combination of preparation or anticipation, response (including detection of an emerging risk), and recovery phases – with a greater or lesser degree of consideration to mitigation approaches to reduce the adverse effects of hazards [20–23]. Similarly, a range of factors have been identified as potentially important contributors to the effectiveness of resilience strategies in response to and recovery from shocks over time [24]. There may be processes of feedback that influence trajectories over time, and elements of learning from previous exposures that affect future response [25].

Clarity of approach concerning the assessment and measurement of health system resilience and community resilience would facilitate the development of strategies that build resilience and facilitate our understanding of the consequences these strategies could have on health and health system outcomes [26]. However, operationalisation is a challenging area of resilience research. Broadly speaking, we can distinguish assessment, which is intended to inform management interventions principally by identifying risks, opportunities and alternative strategies to change (sometimes as a precursor to purposeful transformation); from measurement, which is concerned with early detection of change for situational awareness purposes [27]. Previous literature work has identified four main approaches to operationalisation: the use of [i] qualitative conceptual frameworks; [ii] semi-quantitative indices or metrics of resilience; [iii] conventional quantitative (statistical) approaches; and [iv] systems modelling [28].

A particular challenge to operationalisation is that the rationale for pursuing given approaches to resilience may vary. The purpose of operationalisation may be motivated by either or both of anticipatory needs (e.g. in identifying gaps in preparedness or understanding of particular risks facing community health systems), or action in the event of a shock. There may be differences of approach regarding what to measure and whether the focus should be on established, “hard” metrics (e.g. the number of community health workers) as opposed to “soft” measures that may be relevant to system resilience (e.g. community health worker motivation). Finally, there are likely to be multiple audiences for resilience assessment and/or measurement findings, ranging from national and international stakeholders who may be primarily concerned with long-term surveillance and system strengthening, through to local level actors (e.g. facility managers) requiring data on system resilience for day-to-day operational purposes.

Prior work reviewing approaches to assessment and/or measurement of resilience in community health systems is limited in volume. Initial scoping work identified just one key review, drawing together evidence published to early 2019 [26]. There is, in addition, evidence of a disconnect between the literature on health system resilience – in which the role(s) of communities are frequently overlooked – from literature on community disaster resilience more broadly [13]. However, a large body of primary and conceptual work has been published since the original review was completed, especially in the context of responses to the COVID-19 pandemic. A key purpose of this review was therefore to revisit the literature on community health system resilience measurement, to establish what new lessons we might draw from literature published since the original review was completed.

## Methods

In this review, we sought to answer the following research questions:

1. What assessment approaches and/or metrics to capture resilience in community health systems have been proposed to date?
2. What evidence is there on the utility of these approaches for informing decision-making on resilience-building activities at community level?

Finally, we sought to push beyond analyses presented elsewhere in the literature to outline a tentative set of metrics and assessment dimensions for community health system resilience, as a precursor to future empirical work. Although the driving focus for the review was on resilience to climate hazards, we looked broadly at community health system preparedness, response to, and recovery from a full range of exposures.

### Overview and search strategy

This was a scoping review of literature published since 2019, building on findings published in the earlier Bhandari and Alonge review [26]. In common with the original review [26], we applied a scoping review approach because this is useful for addressing broadly defined research questions and to help understand the nature and extent of current research evidence on a topic of interest [29]. We conducted keyword-structured searches in PubMed and Google Scholar to identify relevant literature published between 01/01/2019 and 05/05/2024, replicating the approach used in the original review – i.e. combining the keywords “community resilience”, “definition”, “indicator”, “framework” and “health system” for a Google Scholar search, and supplementing these terms with the MeSH-enriched term “community health services” for a PubMed search (as no direct equivalent for the term “resilience” used in the original study – and as applied to systems rather than psychological phenomena – now exists in the MeSH tree structure).

### Screening and selection

We applied a series of criteria identify relevant papers for inclusion (see Supplementary Materials S1). Articles were included if the title or abstract included the keywords “resilience”, “community”, “framework”, “definitions” and/or “variables”; the paper in question was specific to public health and health systems; the paper provided some guidance conceptually or operationally on the topic of community resilience; and the research described took place in high-income countries (HICs) and/or LMICs. In line with the original review, we kept inclusion criteria by country status broad to ensure ability to draw lessons from the widest possible range of work on community health system resilience. We also applied considered resilience to the full spectrum of shocks or chronic stressors considered in the literature, ranging from humanitarian crises through natural hazards to long-term challenges to community health system sustainability e.g. chronic funding and health workforce recruitment and retention pressures.

We followed a screening process in line with Joanna Briggs Institute guidance on the conduct of scoping reviews [30]. Manuscripts were independently screened based on title/abstract, and then on full text, by two members of the project team to determine eligibility for inclusion in the review. Both first and second stage screening was performed using Rayyan QCRI, a free web application to support the conduct of systematic reviews by researchers working remotely [31]. Screening outcome disagreements were resolved through discussion with a third reviewer.

### Data extraction and synthesis

Data extraction was performed independently, in duplicate, by all four members of the study team (the final article set being split in half and allocating to two pairs) using a pre-developed proforma. Aggregation of findings was discussed in a series of workshops by members of the project team, following data extraction. Our focus in this analysis was in drawing out (i) operating definitions of community resilience applied in each of the included studies, (ii) domains and/or capacities identified as important for strengthening community resilience, and (iii) mapping conceptual frameworks and metrics relevant to community resilience assessment and measurement as identified in included studies. In line with Scoping Review guidance, formal critical appraisal of included studies was not performed.

In analysing data from included studies, we also gave consideration to sequencing of activities, drawing on the prepare-respond-recover conceptual framing outlined in the introduction. Where specific metrics were identified in the literature these were allocated to specific stages of the mitigate-prepare-respond-recover framework based on discussion within the study team, and set alongside those identified in the original review, for comparison. However, in view of the fact that a majority of metrics outside the prepare-respond-recover elements of the cycle did not truly describe mitigation approaches, this category was instead labelled “ancillary” to better denote the general nature of the measures included.

## Results

**Fig. 1:**
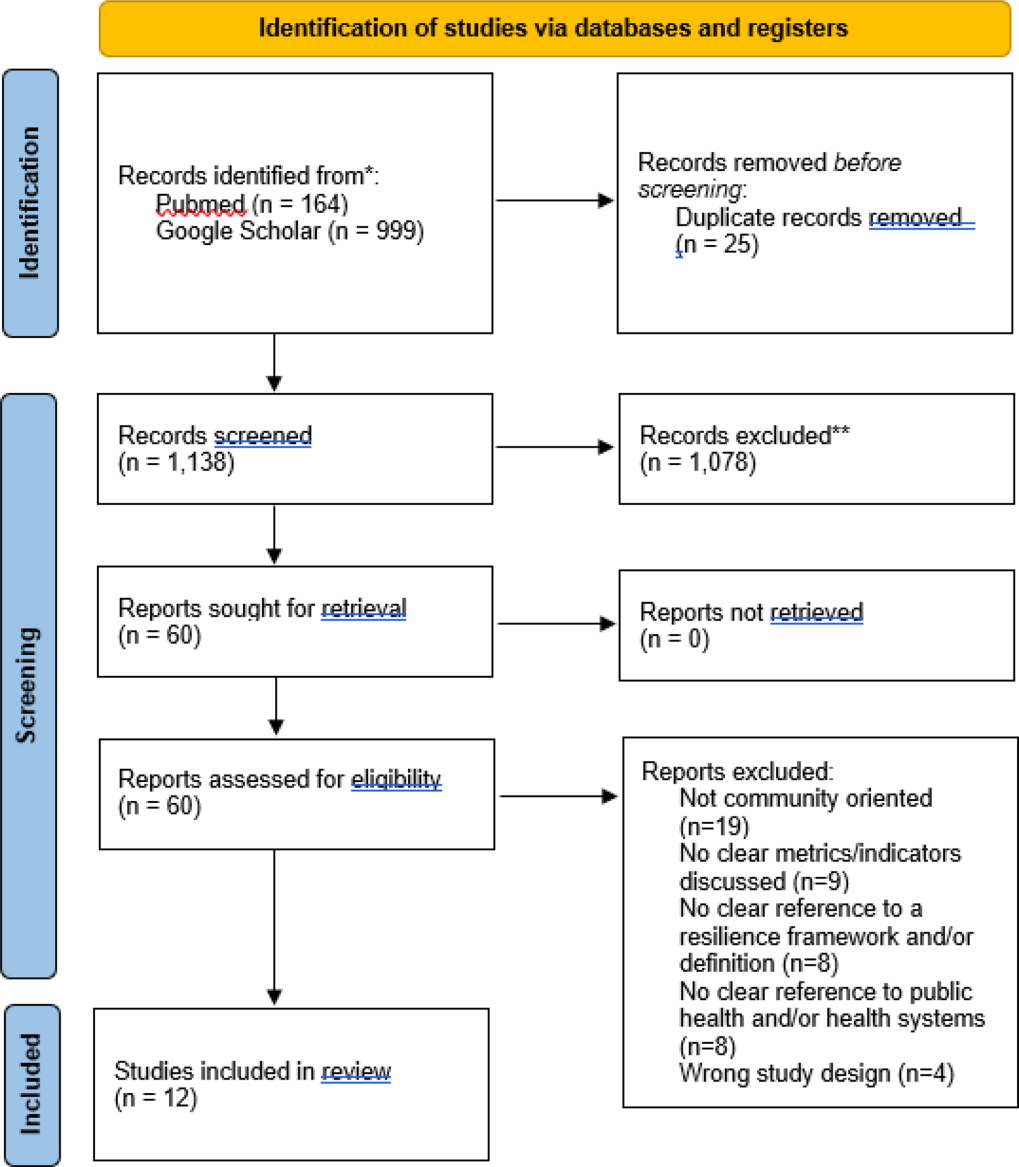
Prisma flowchart describing the process of screening and selection for included studies

A total of *n* = 12 studies were included following screening (see the PRISMA flowchart in Fig. 1). Included studies were diverse in design, including a systematic review (*n* = 1), narrative reviews (*n* = 3), qualitative studies (*n* = 2), mixed-methods studies (*n* = 5) and one quasi-experimental study. Geographical coverage was also broad, from globally oriented studies to focused analyses of system resilience in Cambodia [32], China [33–36] and Pakistan [37]. Five of the included studies explicitly addressed natural and/or climatic hazards [32, 34, 37–39]; all of the remaining 7 papers considered aspects of pandemic resilience through the prism of work on COVID-19. A detailed summary of characteristics and findings from all included studies is given in Supplementary Materials S2.

### Definitions of community health system resilience

Following a similar approach to Bhandari and Alonge [26], we categorized definitions presented in included papers into process-based definitions, absence of adverse consequences definitions, and range of attributes definitions (see Table 1). In the original scoping review [26], definitions discussed were specific to community resilience, with the definition adopted for the review conceptualising community resilience within the broader context of health systems. In the 12 papers considered for this review, the range of definitions and breadth of focus considered was broader, including “health system resilience” [37–40], “community resilience” [33–35, 41], “disaster resilience” [37], “urban resilience” [42], and “public safety resilience” [36]. No systematic differences were identified in definitions applied between those papers focused on natural and/or climatic hazards and communicable disease threats.

**Table 1:** (Modelled after Bhandari and alonge). Definitions of community resilience used in the included studies

Citation/Year	Definition
*Process definitions (Resilience as a dynamic, ongoing process)*	
Fenxia, 2022 [33]	Community resilience as the community’s capacity to maintain, adapt, recover, and improve in the face of the COVID-19 pandemic. It emphasizes the dynamic process of resilience, considering both social and physical factors.
Saulnier et al., 2020 [32]	If a health system is able to integrate and process knowledge, anticipate and cope with uncertainty, manage interactions with other systems at multiple levels (interdependence) and create a socially and contextually accepted system (legitimacy), it is then capable of managing shocks
Shi et al., 2023 [35]	Community resilience is defined as the ability of a community to mitigate and resolve crises in the face of sudden events using its own community resources and protection capacities, guaranteeing the normal functioning of the community’s original functions and quickly recovering from the crisis.
Zhang et al., 2023 [36]	Introduces concept of public safety resilience to explain the public capacity under public emergencies. Community resilience is the ability of the community to resist impacts and recover from them by using community resources during public emergencies.
*Absence of adverse effects definitions*	
Asfoor et al., 2024 [38]	Health system resilience defined as “the capacity of health systems to absorb, adapt and transform when exposed to a shock such as a pandemic, natural disaster or armed conflict. A resilient health system maintains core functions and structure when a crisis hits. In addition, this system learns from lessons learned through the crisis and reorganizes “symptoms” of an approaching crisis”
Rawat et al., 2022 [39]	Health system resilience defined as the “capacity of health actors, institutions, and populations to prepare for and effectively respond to crises; maintain core functions when a crisis hits; and, informed by lessons learned during the crisis, reorganize if conditions require it”
Saulnier et al., 2022 [40]	Resilient health systems have the capacity to absorb shocks using existing resources while maintaining the same essential functions as before, adapt to them by adjusting their functions and use of resources, or fundamentally transform their functions to reduce risks in response to the shock
Suleimany et al., 2020 [41]	Community resilience indicates the capability of people and communities to retain optimal performance in the event of various natural and anthropogenic crises
*Range of attributes definitions*	
Chen and Quan, 2021 [42]	Urban resilience: “Urban areas that can withstand disasters through their own abilities, reduce disaster losses, and reasonably allocate resources to recover quickly from disasters.”
Ma et al., 2023 [34]	Resilience defined as the ability of communities to absorb, adapt, and recover from shocks such as natural disasters and public health crises.
Sajjad, 2021 [37]	Disaster resilience: “the ability to absorb and resist the disturbances (external/internal shocks), the competency of reorganization, fast recovery, and perform better in the future are the common features of resilience across its different definitions
Wang et al., 2021 [43]	Applies as negatively framed definition: “insufficient community resilience” refers to that under the disturbance of external risk, the community cannot actively adapt to and respond to the disaster by integrating internal and external resources, and cannot summarize experience in time after the disaster to improve the effectiveness of crisis governance”

Further difference from the original review may be found in the level/scale considered; while studies included in the original review focused primarily on definitions aimed at the community, household, and individual scale, resilience definitions in our review also include higher governance scales; e.g., urban areas [42] and interdependencies across multiple systems and levels in a specific context [32]. Similar to definitions outlined in Bhandari and Alonge (2020), definitions in studies included in this review emphasized systems ability to absorb, adapt, recover [36, 37, 43], and at times, transform [40], therefore seeing resilience as a dynamic process. Finally, the definitions clearly point to a multi-dimensional nature of resilience, as definitions outline economic [42], institutional [39] and social [32] dimensions.

For the 12 definitions considered in our review, we see a shared emphasis on the key components of resilience, i.e., absorbing (maintaining similar levels of health service delivery using similar resources), adapting (delivering similar levels of health service with fewer or different resources), and transforming (changing health system structures and/or functions to respond to a new environment) [33, 35, 38–40]. There is also a shared understanding of the importance of maintaining the core functioning under crisis [33, 38, 40]. Several definitions focus on transformation and learning [32, 40], outlining the importance of transformation in building or enhancing resilience. Differences were primarily in terms of the scope of resilience considered; as mentioned, while some focus on health system resilience [32, 38–40], others focus on community resilience [33–35, 41] or a broader resilience, such as disaster [37], urban [42], or public safety [36]. Furthermore, while most studies take a positive stance of resilience, Wang and colleagues define resilience in a different manner by focusing on the negative aspects and failure to be resilient [43].

### Conceptual frameworks for community health system resilience

Bhandari and Alonge identified three key conceptual frameworks unifying papers included in the original review – none of which emerged from the health or health systems literature [44–46]. Papers included in this review referenced a series of frameworks, some drawing on one or more of the frameworks given above [34], others originating elsewhere in the literature in health systems [32, 40], some developed by study authors for their analyses but nevertheless focused principally on health and community health systems [33, 34, 43], and a small number of studies referencing frameworks grounded in public safety [36]. Table 2 summarises key elements or capacities contributing to community health system resilience identified across the included studies, within each of these conceptual frameworks. Once again, no systematic differences were identified between those studies exploring resilience to natural and/or climatic hazards, and those concerned with communicable disease threats.

**Table 2:** Elements of community resilience identified in included papers

Elements	Description
Local Knowledge	-Integration of traditional and indigenous knowledge [34, 39] -Capacity building and skill development for emergency preparedness [33, 35] -Participatory action research and planning to address vulnerabilities [34] -Continuous learning and feedback loops to improve disaster response [34, 35] -Incorporation of cross-sectoral knowledge exchange [35, 39]
Community Networks and Relationships	-Strong social cohesion, trust, and reciprocity [33, 34, 41] -Mobilization of community groups and collective action [33] -Informal and inter-organizational support networks [34, 40] -Bridging and linking networks for broader societal collaboration [34, 40, 41] -Civic participation and community leadership [34, 41]
Communication	-Transparent and culturally appropriate messaging [34] -Use of traditional and social media for real-time information dissemination [40] -Incorporation of diverse information sources for resource distribution and coordination [40]
Health	-Pre-existing robust health systems and healthcare delivery [40, 41] -Resources for both short-term and long-term care [41] -Promoting individual health resilience (e.g., hygiene, education, and well-being) [41]
Governance	-Transparent decision-making and inclusive processes [39–41] -Strong government leadership and decentralized decision-making [39–41] -Cross-sectoral collaboration and conflict resolution mechanisms [39, 41] -Long-term vision and planning for adaptive capacity and empowerment [39, 41]
Resources [33, 34, 39, 41]	-Availability of financial, technical, and social resources. -Climate-resilient and green infrastructure [34, 41] -Supply chain continuity and access to medical equipment and basic goods [41] -Decentralized systems for efficient resource distribution [41] -Community-level training, emergency drills, and recovery plans [34]

Frameworks adopted in the included studies fell broadly into three categories. Some studies focused on organisational or meso-level characteristics contributing to system resilience, especially where applying frameworks developed originally to describe macro-level health system activities. For example, two studies by Saulnier and colleagues drew on an analytical framework for resilience governance focused on contributors to absorptive, adaptive and transformative capacities [32, 40]. Similarly, Asfoor and colleagues identified a series of pre-requisites or system enablers (”antecedents”) for health system resilience, resilience attributes that spanned features such as availability and flexibility of funding, adaptative and transformative capabilities, learning and advocacy, and leadership fostering of innovation, creativity and diversity [38].

A second set of studies considered community-level characteristics often with a focus on aspects of social capital. For example, Fenxia explored the relationship between participation in disaster risk management activities and the indicators on the Community Advancing Resilience Toolkit (CART), which included five, asset-based dimensions spanning markers of connection and caring, community resources, transformative potential (including collaborative working and learning culture), community-level disaster management, and information exchange locally [33]. Work by Ma et al. drew on Norris’ categorisation of components contributing to resilience, spanning social capital and local knowledge systems, through to preparedness and response capacity, and adaptive infrastructure and resources (e.g. green infrastructure and ecosystem services, financial systems and decentralised and distributed systems) [34].

A final set of studies considered community assets within the context of wider regional or national architectures supporting local level resilience. These encompassed aspects of social (community resources), organisational, infrastructural and wider economic resilience (including regional economic development) [35], or multi-level approaches taking into account dimensions of personal resilience, alongside those of the community within which people are embedded (including demographic and financial factors, management of people and funds, and improvement capability in local institutions) and government regionally and nationally [36].

### Resilience metrics

Of those studies included, some (but not all) identified potential metrics to support measurement of health system resilience at community level. We collated proposed metrics across both the Alonge and Bhandari review and those papers included in this review to produce a combined set, mapped against (i) the elements identified in Table 2, and (ii) the prepare-respond-recover analytical framework outlined in the introduction. A full list of these metrics and their source studies is given in Table 3.

**Table 3:** Full list of putative indicators of community health system resilience identified in this scoping review

Element	Phase			
	Ancillary	Prepare	Respond	Recover
Local knowledge	-Baseline awareness of hazards and associated risks [41]*	-Proportion of community that have participated in evacuation drills [33]* -Proportion attending disaster education [33]* -Existence of a community risk and vulnerability assessment and/or risk register [34]* -Existence of a community hazard map [34]* -Existence of land use plans that have been developed with reference to local hazard and risk assessment(s) -Location and type of responsible agencies/institutions/offices for implementation of response actions during a shock -Existence of community contingency plan(s)	-Presence of monitoring and evaluation plan for community contingency plans [34]* -Frequency of review of risk and vulnerability assessment during the response phase [41]*	-Integration of lessons from monitoring and evaluation to inform future practice [34]*
Networks and relationships	-Number of community meetings [34]* -Number and diversity of partnerships between community organisations [34]* -Metrics of intra- and inter-community trust [41] -Participation by type of NGOs, civil society volunteers and the private sector in community platforms -Proportion of community members actively engaged in community-based associations and/or events -Proportion of community members willing to provide food and/or monetary support to non-family members -Percentage of workers for community-based organisations and groups that are volunteers -Number of at-risk individuals included in formal and informal networks	-Hazard preparedness and response capacity building programmes in place [34]* -Proportion of female-headed households and those from marginalised groups involved in local planning processes -Measured participation of at-risk groups in risk assessment processes	-Proportion of community members participating in volunteer responder groups [33]*	
Communication	-Proportion of the population with a mobile phone [37]*	-Presence of an Early Warning System -Availability of robust and extended communication network throughout areas at risk	-Deployment of tailored information/communications assets to at-risk populations during the response phase [40]* -Timeliness of communication to at-risk populations during the response phase [41] -Timeliness and comprehensiveness of response to misinformation in the response phase [36] -Proportion of at-risk populations reached by Early Warning System information/alerts	
Health	-Number of health facilities [42]* -Number of inpatient beds [42]* -Typology of emergency services [35]* -Number of health professionals, by cadre, in the community health system [42]* -Trust in local health services [40]* -Community access to essential health services (and equity in this access) [41]* -Mortality and morbidity from key marker diseases [41]* -Self-rated wellbeing [41]* -Percentage of households covered by basic medical insurance [41, 42]*	-Proportion of health workforce trained in preparation for and management of responses to relevant hazards [38]* -Existence of a long-term plan for investment in improvement and sustainability of local health services [38]* -Scale and scope of surge emergency service capacity in the event of a disaster [41]	-Establishment of an emergency service leadership group (for response coordination) [35]* -Timeliness and efficiency of emergency service deployment to affected areas [41]*	
Governance	-Perceived legitimacy of local governance systems [40]* -Perceived transparency of local governance systems [40]* -Inclusivity in local governance systems [40]*	-Community confidence in government agencies and other state actors to mitigate damage during and after an event [41]* -Existence of land-use plans that have been subject to formal consultation processes	-Community confidence in government agencies and other state actors to mitigate damage during and after an event [41]* -Timeliness and efficiency of government and/or agency response (by response dimension) [41]	-Perceived level of control/influence over community preparedness and response based on prior learning and experience
Resources	-Proportion of property-owning households [37]* -Household dependency ratio [37]* -Year-end balance of savings per household [42]* -Availability and extent of access to critical resources e.g. telephony, domestic piped water supplies, domestic gas supplies [37, 41]* -Road network density [42]* -Total annual utility supply per household (water, electricity, gas etc) [42]* -Green space [34, 42]* -Poverty rate (e.g. UNDP multi-dimensional poverty index) [37]* -Proportion of working-age adults in the community who are employed [37]* -Female labour participation rate [37]* -Child labour rate [37]* -Youth literacy rate for males and females [37]* -Markers of local economic performance (e.g. total regional tax revenue, annual expenditure per capita budgeted by the government to the community) [35, 37, 42]* -Highway freight volume [42]* -Industrial wastewater discharge/sulphur dioxide emissions [42]* -Percentage of households with year-round access to clean water -Proportion of household heads with secondary education or higher -Number of civic organisations locally/10,000 population	-Proportion of households with a basket of defined emergency supplies [33]* -Diversity of funding sources for response work [38]* -Presence (and typology) of emergency services at community level -Disaster relief funding per capita	-Timeliness and efficiency of broader emergency service mobilisation during the response phase [36]* -Community fund-raising capacity and capability [36]* -Capacity and capability to absorb and distribute newly mobilised funding during the response phase [36]*	

Indicators identified from new literature included in this review are denoted with a *; those from the original review are in plain black text [26]

**Fig. 2:**
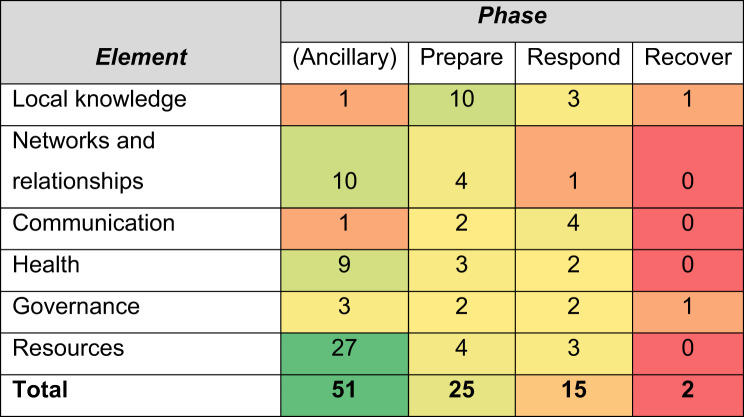
Distribution of community health system resilience measures identified in included studies, by domain. Figures given indicate the total number of putative indicators identified across all included studies, not the total number of studies (source: author derived)

As shown in Fig. 2, a majority of indicators did not relate to any of the dynamic elements of the prepare-respond-recover framework, but were instead concerned with ancillary characteristics or markers at community level that may or may not be associated with resilience in response to shocks. Measures in this category included metrics such as the proportion of working adults in the labour force and female labour force participation rate [37], markers of local environmental contamination such as industrial wastewater discharge levels [42] and markers of networking at community level such as the presence and type of partnerships between community-based organisations locally [34] – among others. Metrics in this group also included many focusing on local health systems, spanning simple measures of capacity (e.g. number of health facilities, number of inpatient beds [42]) and service diversity (e.g. the typology of emergency services available locally [35] and breakdown of the health workforce by cadre [42]). A number of proposed metrics also considered background population health including mortality and morbidity rates, and self-rated wellbeing, as proxies for vulnerability to adverse health effects from shock exposure [41].

Indicators relating to the prepare phase frequently considered aspects of local knowledge, including community participation in forms of disaster education and training, and involvement in evacuation drills [33]. Other markers included the presence of community risk and vulnerability assessments, or hazard maps and related preparatory activities such as simulation exercises to model out responses to known hazards [34]. A number of measures considered networking and relationships – such as the presence of hazard preparedness and response capacity building programmes [34], or community resources including diversity in funding sources to support preparedness and response [38], and the percentage of households in the community with a (undefined) basket of emergency supplies [33].

Response indicators covered all of the elements outlined in Table 2. Indicators in the communication domain included markers relevant to equity, such as deployment of tailored information and communication assets to populations defined as being at-risk [40] and timeliness of that communication [41]. Other indicators included governance markers such as the timeliness of government agency responses during this phase of the cycle, and indicators of community confidence in that response [41], and metrics of capacity and capability to mobilise resources locally, such as the extent of local fund-raising, and capacity or capability to absorb and allocate funding during the response phase [36].

Only one of the studies included in the updated review identified measures relating to post-event recovery and learning. In this case, the focus was on local knowledge, and specifically the integration of lessons from monitoring and evaluation to support improvements in practice [34].

## Discussion

We identified a set of 93 candidate indicators for community health system resilience, 73 of which were derived from papers included in this review, and 20 from the original scoping review that our analysis has updated. Putative indicators overwhelmingly focused on contextual factors and aspects of preparedness at community level, with few addressing dynamics of response and recovery – although some did consider aspects including timeliness and scale/scope of action during the response phase (for Early Warning Systems communication, and emergency response activity for example). Importantly, no systematic differences were identified in resilience definitions, conceptual frameworks applied, or resilience indicators put forward between those papers concerned with natural and/or climatic hazards and those addressing communicable disease threats (in this analysis, COVID-19).

Differences in reported findings between studies more obviously reflect the diversity of disciplinary perspectives on which individual studies drew, spanning health systems [32, 40], community resilience more generally [43], and some from disaster risk reduction [33, 41]. This can be seen in the range of definitions of community health system resilience – many presenting views of resilience that spanned some combination of process, absence of adverse effects, range of attribute approaches, or all of the above. Analytic lenses applied were similarly diverse, with some studies embedded firmly within the health system resilience literature [32, 40], other work concerned with aspects of public safety management [36].

Candidate measures of community health system resilience identified in this updated review overwhelmingly focused on non-specific items that may – collectively – give a view of vulnerability at community health system level but which do not meaningfully relate to mitigation to reduce future hazard-related risks. In addition, engagement with the extensive, existing literature on community vulnerability assessment to inform the selection of these measures appeared to have been limited and it was often unclear on what conceptual basis they had been included in the relevant papers as potential resilience indicators [47, 48]. For this reason, we collectively labelled these measures “ancillary”. In our view, testing the sensitivity and specificity of these measures in practice should be a priority for future work to determine what – if any – value they have as indicators of system resilience.

Where proposed measures did address dynamic features of resilience, most considered preparedness and very few considered system behaviours in the recovery and/or learning phase of the disaster risk management cycle. Measures were also concerned exclusively with measurement of absorption or adaptive response – and not in any discernible way with transformation as a resilience strategy. This likely reflects continuing conceptual challenges in describing what transformation in the context of health systems in general may look like [28].

Finally, important gaps were identified in topic coverage in included studies. First, none of the included studies directly addressed equity considerations in community health system resilience beyond general and non-specific criteria such as measures of inclusivity in governance arrangements, and equity of access to health services. Many of these measures were ancillary rather than falling within the mitigate-prepare-respond-recover cycle, although some studies did engage with – for example – the deployment and timeliness of communications messaging to groups identified as being at risk [40, 41]. One possible reason for the lack of attention is the relative absence of discussion of health outcome measures – although variations in outcomes for key indicator diseases did feature in one study [41]. Tangential discussion of equity likely also relates to enduring conceptualisation of resilience as a normative concept in much of the health systems literature, and insufficient attention to the power dynamics that may determine how and why community health systems respond to different types of shock in the ways that they do [3].

Other gaps included a strong service supply focus for measures in the health domain, without attention to changes in care-seeking behaviour that may occur among community members following an event (and how these may differ between groups). Finally, there was a strong focus on public and third sector service provision, with only one paper considering the role of the private sector as a potential contributor to community health system resilience [40].

Overall, a striking finding from this updated scoping review is that – notwithstanding the rapid rise in research interest in health system resilience in recent years – analysis of community health system resilience remains under-developed. In addition, although we did not formally critically appraise included studies (in line with the conventional scoping review approach), many included studies had clear and significant methodological limitations. None of the included studies assessed resilience with respect to a specific health outcome(s). Of 12 studies included, 8 were empirical analyses [32, 33, 35–39, 42, 43] and though some of these papers put forward candidate measures of resilience, the conceptual basis on which measures were selected was often unclear. None of the papers empirically tested the basket of 20 indicators identified in the original review, so questions concerning the validity of these measures remain unaddressed [26]. In addition, while some studies presented aggregate resilience scores at single, cross-sectional time points [33, 37], no longitudinal analyses were attempted in any of the included studies to determine the validity of proposed metrics in capturing changes in key community health outcomes over time. This points to an urgent need for field testing of community health system resilience assessment and measurement approaches, to evaluate the extent to which proposed measures adequately capture dynamic aspects of preparedness, response and recovery in response to public health threats.

There are important limitations to the analysis presented here. Database coverage was limited – in alignment with the original review. There are in addition recognised challenges to replicability of searches using search engines such as Google Scholar because of tailoring of returns based on user preferences [49]. Nevertheless, for the purposes of consistency with the earlier review on which this study builds, we applied the same search strategy as was deployed there [26]. Standard limitations – including of potential publication bias – apply, and in addition our findings may have been influenced by a focus on English language-only publications, especially given the rapid expansion of the literature on resilience during and after the pandemic.

A potentially important conceptual limitation concerned the application of an existing theoretical framework (prepare-respond-recover) to organise findings and the list of putative indicators identified. The outlines of this framework are well recognised in the DRR literature and have been extensively applied to support analysis in that field; they are also core components of recognised health systems frameworks including the WHOs emergency cycle and the WHO operational framework for building climate resilient and low-carbon health systems [50, 51]. While the closed nature of the cycle described by this framework has been critiqued by some authors [52], this has typically been on the grounds that it does not take full account of dynamism in disaster risk management. Given the striking absence of dynamic measures identified in this review (see above) it is unlikely that use of a different conceptual framework would have better drawn these out.

A firm recommendation from this review is that researchers and practitioners working on community health system resilience should now formally evaluate the practicality and utility of measures within the set of 93 candidate indicators identified above. There is a need to build consensus around measures that are sensitive and specific enough to support decision-making in different settings, and to enable evaluation of the extent to which the burden of preparedness, response and recovery work in relation to climate hazard exposures falls inequitably on different groups within community health systems. However, any such evaluation work will need to acknowledge that formal, quantitative measurement is only ever likely to form part of broader, mixed-methods approaches to monitoring resilience [27].

## Electronic supplementary material

Below is the link to the electronic supplementary material.

Supplementary material

## Data Availability

All data generated or analysed during this study are included in this published article [and its supplementary information files].
