# Supplementary material for: Assessing and measuring community health system resilience – an updated scoping review of approaches
Source: BMC Health Serv Res. 2025 Dec 4;26:34. doi: 10.1186/s12913-025-13802-6 (PMC12781277; doi:10.1186/s12913-025-13802-6)
Supplement: Supplementary file 1 — Supplementary material [file 12913_2025_13802_MOESM1_ESM.docx]

Supplementary materials S1. PICO structure for the review

| ***Domain*** | ***Included*** | ***Excluded*** |
| --- | --- | --- |
| Population | All human populations from low- and middle-income settings (defined using World Bank country lending criteria), and primary healthcare facilities in these settings | Literature on secondary and tertiary referral centres as these are not regarded as “community-based” in the same sense as primary care facilities. |
| Exposure | Climate-related hazards but with a specific focus on extreme heat and flooding |  |
| Intervention | This review will not consider interventions – the focus is on assessment and measurement approaches at community level (up to the level of districts/municipalities).  Broad definitions:  **Assessment:** intended to inform management interventions principally by identifying risks, opportunities and alternative strategies to change (sometimes as a precursor to purposeful transformation)  **Measurement:** concerned with early detection of change for situational awareness purposes | Studies in which no formal metrics are proposed and/or no formal assessment criteria/dimensions are set out.  Studies in which the primary focus is macro- (national health systems) or meso-level (regional health systems or other levels down to municipality/district). |
| Comparator | Dependent on study design |  |
| Outcome | "Resilience" - however defined by the study |  |
| Languages | English only | Any non-English language |
| Database coverage | Peer reviewed literature: PubMed, GoogleScholar |  |
| Date of publication | Studies published since September 2019 | Any output preceding this publication period |
| Study designs | - Systematic reviews/MAs - All experimental and quasi-experimental designs - All observational designs - Narrative and conceptual reviews - Modelling studies | - Case series and case reports - Letters - Commentaries and editorials - Conference abstracts |

**Supplementary Table S1. Inclusion and exclusion criteria applied in the scoping review.**

Supplementary materials S2. Table of details for all included studies

| ***Study*** | ***Setting(s)*** | ***Exposure/hazard*** | ***Design*** | ***Population*** | ***Resilience definition*** | ***Outline conceptual approach/framework used*** | ***Metrics and/or indicators considered*** | ***Health outcomes considered*** |
| --- | --- | --- | --- | --- | --- | --- | --- | --- |
| Asfoor et al (36) | Global literature review - no specific context examples discussed in paper | Not explored; referred to as "crises" | Narrative review | N/A | "the capacity of health systems to absorb, adapt and transform when exposed to a shock such as a pandemic, natural disaster or armed conflict. A resilient health system maintains core functions and structure when a crisis hits. In addition, this system learns from lessons learned through the crisis and reorganizes “symptoms” of an approaching crisis" | "Framework of health system resilience" developed from analysis, which distinguishes: **1. Antecedents (**prerequisites for resilience) e.g. decentralisation, funding and resources, staff environment and motivation, integration and networking;  **2. Attributes** e.g. funding availability and flexibility; adaptation; transformation; learning and advocacy; and progressive leadership  **3. Consequences** (expected results of a resilience health system), such as improved health system performance and improved health coverage overall | Inferred, focused primarily on supply-side aspects and including: - transferred power and responsibility for decisions - long-term plan for funding and resources - adaptive staffing, workforce training and attention to well-being - redundancies - multiple funding and inputs sources - flexibility to reallocate funds and resources - able to move responsibilities | N/A |
| Chen et al (40) | Yangtze River Delta in China between 2003-2017 | Covid-19 | Mixed-methods study (e.g. evaluation) | N/A | Urban resilience: "urban areas that can withstand disasters through their own abilities, reduce disaster losses, and reasonablly allocate resources to recover quickly from disasters" | Mathematical model to evaluate urban resilience using TOPSIS ranking method.  Indicators for model derived from 4 subsystems relevant to urban resilience spanning: 1. Economy: diversity and expenditure can cushion against impacts on economy and help with recovery speed.  2. Ecology: green spaces help prevent disease transmission, improve wellbeing and health. 3. Infrastructure: carrying capacity for health response to Covid. 4. Social systems: represents response and direction of development post-Covid. | **Multiple, spanning**  **1. Economic** e.g. annual GDP, expenditures budgeted by local governments, total regional tax revenue (financial resources of the urban area) **2. Ecological e.g. g**reen area (self-repair ability of the urban area), industrial sulfur dioxide emissions (pollution status of the air) **3. Infrastructure** e.g. number of hospitals and health centres, number of beds in hospitals and health centres, road area and power supply  **4. Social policy resilience** e.g. population density, unemployment rates, healthcare staffing levels in different parts of the system | "Health" in general, with some upstream effects eg less air pollution |
| Fenxia et al (31) | Wuhan, Chengdu and Shanghai, China. Data collected during Feb/Mar 2020. | Covid-19 | Mixed-methods study (e.g. evaluation) | Survey: 650 residents in the three cities. 85% under age of 50. 66% women, 60% unmarried, 72% college educated. 45% living in cities for 10+ years | Community resilience: "a community's ability (perceived by the residents) to cope with risks" | CART (Community Advancing Resilience Toolkit) score – spanning 5 domains with 24 core items ranked from 1 to 5 spanning: 1. **Connection and caring**: as measures of community social capital 2. **Resources**: in terms of material resources, but also human capital e.g. leadership quality  3. **Transformative potentia**l: and relevant supporting assets within the community  4. **Disaster Management**: spanning preparedness and response  5. **Info and Communication**: in terms of range and quality of communications, and the degree of public trust in the messages being imparted | Markers influencing the CART score included metrics of  1. Participation (e.g. respondent household h**as emergency supplies, respondent has attended disaster training and/or has participated in evacuation drills** 2. Communication and p**articipation in volunteer responder groups** 3. Background and/or demographic factors including city of residence, gender, education, age, marital status | N/A |
| Ma et al (32) | Global lit review - no specific context examples discussed in paper. Papers from North America (n=12), Europé (10), Asia (6), Africa (4), Oceania (3) | Natural disasters and public health 'issues' --- floods, earthquakes, hurricanse, wildfires, Covid, Ebola, influenza, NCDs | Narrative review | N/A | None given. | "One Community at a Time" Framework, spanning: **1. Social capital and networks 2. Local knowledge and learning 3. Effective governance and leadership 4. Preparedness and response capacity including early warning systems, emergency plans and protocols etc 5. Adaptive infrastructure and resources including climate resilient infrastructure, access to adequate financial resources and other prerequisites** | Multiple spanning multiple domains including:  1. Assessing community vulnerability and risks through, for example, hazard maps  2. Engage and mobilize community members – through e.g. frequency and extent of participation in community meetings  3. Develop a community resilience plan  4. Implement resilience-building initiatives – and measurement of progress towards goals set out in these 5. Build partnerships and networks – including the number and diversity in partnership structure  6. Foster local knowledge and learning, through training and education 7. Enhance preparedness and response capacity, through e.g. emergency drills and simulations. Allocation of resources for preparedness and response | N/A |
| Rawat et al (37) | Three drought-affected regions of Ethiopia | The 2015/ 2016 drought | Qualitative study | Community-level health system stakeholders including health workers, community leaders, NGO representatives and others | Health system resilience per Kruk definition: "capacity of health actors, institutions, and populations to prepare for and effectively respond to crises; maintain core functions when a crisis hits; and, informed by lessons learned during the crisis, reorganize if conditions require it” | No specific resilience framework used, instead emphasizing health system performance and bottlenecks. | Inferred from the analysis and include measures relating to: **1. Organized community groups linked to the health system**  **2. Adaptable human resource structures and service delivery models** 3. **Training and preparedness 4. Strong government leadership with decentralized decision-making** | N/A |
| Sajjad et al (35) | All districts in Pakistan, except Gilgit Baltistan, Azad Jammu, Kashmir, and the Federally Administered Tribal Areas | No specific hazards - disasters in general | Quasi-experimental study (e.g. ITS) | Secondary data for community and household-level indicators | Disaster resilience: "the ability to absorb and resist the disturbances (external/internal shocks), the competency of reorganization, fast recovery, and perform better in the future are the common features of resilience across its different definitions" | No specific frameworks or approaches - indicators selected from well-established domains of disasters resilience (economic, institutional, social) | Multiple, spanning  1. Economic e.g. poverty rate, extent of home ownership, labour force participation measures 2. Institutional e.g. access to improved drinking water and electricity, access to transport, literacy rates between male and female adolescents 3. Social e.g. total population, urbanisation rate, sense of place in the local community | N/A |
| Saulnier et al (30) | Cambodia, eight villages in two districts | Flooding | Qualitative study | Community members and leaders; | Not given. | Dimensions of Resilience Governance Framework by Blanchet et al. The framework posits that if a health system is able to integrate and process knowledge, anticipate and cope with uncertainity, manage interactions with other systems at multiple levels (interdependance) and create a socially and contextually accepted system (legitimacy), it is then capable of managing shocks. | Inferred from the analysis and include – among others – measures relating to:  1. Transport and communications e.g. good road access, availability of mobile phones  2. Health workforce considerations including access to skilled birth attendants, access to community-based skills for pregnancy and childbirth  3. Community capacities including having local coping capacities (e.g., stockpiling food and supplies, preparing transport, preparing a clean water source and saving money) | N/A |
| Saulnier et al (38) | Global | Covid-19 | Narrative review | NA | Resilient health systems have the capacity to absorb shocks using existing resources while maintaining the same essential functions as before, adapt to them by adjusting their functions and use of resources, or fundamentally transform their functions to reduce risks in response to the shock. | Dimensions of Resilience Governance Framework by Blanchet et al. Systems must be able to integrate, process, and make decisions using knowledge about their resources, risks, and health needs by interacting with different actors and groups inside and beyond the health system. They are able to anticipate and cope with uncertainty through the actions and decisions of individuals, groups, and networks in response to the shock. They must also be able to manage interactions with other systems beyond the health system (interdependence) and recognize the impact of contextual and external factors on the system’s behavior, capacities, and resources. Finally, systems must create a legitimate system that is trusted to provide socially acceptable and contextually appropriate care. | Inferred indicators based on necessary attributes by domain:  1. Knowledge: e.g. need to quickly access, use, and integrate information from other systems to improve resilience; need for tailored information to inform varied decisions; ability to monitor risks from beyond the health system 2. Managing uncertainty: e.g. flexibility in resource generation and use; bottom-up processes for decision-making 3. Legitimacy: e.g. power for populations and communities to influence health sustem  4. Interdependence: markers of working work collaboratively; leadership and communication to strengthen collaboration and coordination between stakeholders | N/A |
| Shi et al (33) | China | Covid-19 | Mixed-methods study (e.g. evaluation) | 20 towns and cities in China | Community resilience defined as “the ability of a community to mitigate and resolve crises in the face of sudden events using its own community resources and protection capacities, guaranteeing the normal functioning of the community’s original functions and quickly recovering from the crisis. | Author-developed research framework for community epidemic prevention and control capacity, including four types of resilience:  1. Infrastructure (infrastructure completeness)  2. Organizational (community self-organizing ability)  3. Social (redundnacy of community resources), and  4. Economic resilience (stability of regional economic development). | Indicators outlined by domain, including  1. Infrastructure completeness: e.g. the soundness of epidemic prevention engineering facilities (emergency isolation sites, epidemic prevention inspection stations, safety signs etc); medical and healthcare facilities and medical professional teams and their availability  2. Organizational resilience e.g. the establishment of emergency leadership groups for epidemic prevention and control.  3. Social resilience e.g. access to epidemic prevention funds, sufficient supply of community volunteers  4. Economic resilience e.g. markers of local economic output | N/A |
| Suleimany et al (39) | Global | Covid-19 | Systematic review/meta-analysis | NA | Community resilience indicates the capability of people and communities to retain optimal performance in the event of various natural and anthropogenic crises. | Five-dimension framework to explain community health system resilience:  I- Environmental and Infrastructural,  II- Institutional,  III- Public Health and Well-being, IV- Social, and  V- Economic | Indicators outlined by domain e.g.  1. Institutional Resilience: e.g. markers of efficient leadership and management, institutional preparedness 2. Social Resilience: markers of social trust, social solidarity, safety and protection, social capital;  3. Economic Resilience: e.g. local economic development, supply chain resilience, employment and activity, food security, insurance and financial readiness for shocks 4. Built Environment and Infrastructural Resilience: e.g. transportation, hospital capacity, local housing capacity etc 5. Demographic markers e.g. life expectancy, quality of life | N/A |
| Wang et al (41) | China | Covid-19 | Mixed-methods study (e.g. evaluation) | 14 cities in 7 provinces, 2345 questionnaires and 71 interview data | Negatively defined: "insufficient community resilience” refers to that under the disturbance of external risk, the community cannot actively adapt to and respond to the disaster by integrating internal and external resources, and cannot summarize experience in time after the disaster to improve the effectiveness of crisis governance" | Developed an analytical framework of the formation mechanisms of weak resilience of the community public helth crisis governance, based on the Complex Adaptive Systems (CAS) theory. | - Some indicators can be inferred from Figure 4. For example": access to communities, communities reaction speed to emergencies, risk understanding, administrative management of communities, existence of social organizations in the community. | N/A |
| Zhang (34) | Beijing, China | Covid-19 | Mixed-methods study (e.g. evaluation) | Survey delivered to 980 community members in a district of Beijing | Community resilience is the ability of the community to resist impacts and recover from them by using community resources during public emergencies. | A three-level public safety resilience framework described that includes personal, community, and government resilience, and which measures the resistance and recovery ability. Community resilience is at the core and includes inherent ability (demographic factors, facilities and materials, organization and institution, financial ability), operational ability (people management, materials management, funds management, and public opinion management), and improvement ability (improvement of materials and facilities, improvement of organizational systems etc) | Indicators by domain:  1. Inherent ability - including: - Demographic factors e.g. population structure and characteristics of the community  - Facilities and materials: e.g. the supply capacity of food, water, electricity  - Organization and institution: e.g. emergency plans and daily drills for various emergencies  - Financial ability: the employment, income, investment, and savings of the community residents; the fianncial expenditure that the community can use for public security and the funds for emergency rescue. 2. Operational ability: spanning people management, management of materials and funds management; and public opinion 3. Improvement ability: spanning materials and facilities, improvement of organizational systems and other factors  4. Dimensions of personal resilience e.g. economic and social background; knowledge and skills; levels of family support | N/A |
